# Prediction models for nurse turnover: A protocol for a systematic review

**DOI:** 10.1371/journal.pone.0354384

**Published:** 2026-08-25

**Authors:** Yujia Huang, Chaojun Shan, Xueni Xiao, Chuanya Huang

**Affiliations:** 1 Department of Neurosurgery, West China Hospital, Sichuan University/West China School of Nursing, Sichuan University, Chengdu, Sichuan, China; 2 Department of Obstetrics Nursing, West China Second University Hospital, Sichuan University, Chengdu, China; 3 Key Laboratory of Birth Defects and Related Diseases of Women and Children (Sichuan University), Ministry of Education, Chengdu, China; 4 Department of Nursing, West China Hospital, Sichuan University/West China School of Nursing, Sichuan University, Chengdu, Sichuan, China; Stamford Health System: Stamford Hospital, UNITED STATES OF AMERICA

## Abstract

**Introduction:**

High nurse turnover rates pose significant challenges to healthcare systems, affecting patient care quality, workforce stability, and healthcare institutions’ financial performance. While several prediction models for nurse turnover have been developed, their quality and performance have not been systematically evaluated, limiting their application in practice and making policy. This study aims to systematically review these nurse turnover prediction models and provide valuable insights for healthcare administrators and policymakers to improve nurse retention and optimize workforce management.

**Methods and Analysis:**

This study is a systematic review protocol. We will conduct a comprehensive search of PubMed, Embase, Web of Science, SinoMed, CINAHL, Cochrane Library, CNKI, Wanfang Data, and VIP databases, covering all relevant articles published from the inception of databases through May 31, 2026.Studies that developed prediction models for nurse turnover (with or without external validation) will be eligible for inclusion. Two independent reviewers will search, select, extract, assess, and analyze potentially relevant studies. The Prediction Model Risk of Bias Assessment Tool (PROBAST) will be used to evaluate the quality and risk of bias in the included studies. Disagreements between the two reviewers will be resolved through discussion or consultation with a third reviewer. A narrative synthesis will be conducted to present the characteristics and performance of the models in the included studies.

**Ethics and Dissemination:**

Ethical considerations are not applicable as this is a systematic review of published studies. The results will be published in a peer-reviewed journal.

**PROSPERO Registration:**

PROSPERO Registration Number CRD42024576727

## Introduction

Nurses play a vital role in promoting health, improving health outcomes, and enhancing overall well-being [1]. They not only provide basic patient care but also help formulate health policies and promote primary healthcare, which ensures the sustainability of global health systems [2]. *The WHO Global Strategic Directions for Nursing and Midwifery (2021–2025)* calls for educating enough midwives and nurses to meet population health needs [3]. It also emphasizes creating job opportunities to recruit and retain these professionals where they are most needed. However, the WHO estimated that by 2030, the shortage of nurses will reach 4.5 million, especially in Africa, Southeast Asia, and Latin America, due to factors such as workforce migration, aging populations, and inadequate investment in nursing education and training [4]. Therefore, additional efforts are required to alleviate the current shortage of nurses.

One of the key drivers of nurse shortage is the high turnover rate among nurses, which worsens the crisis in healthcare staffing [5]. The turnover of nurses not only impacts the continuity and quality of patient care but also imposes significant economic costs on healthcare institutions [6]. Franklin et al. pointed out that the pressure from the fast-paced work environment may lead to high nurse turnover in many medical institutions, and the costs associated with nurse turnover can have a huge impact on the hospital’s profit margin [7]. The 2019 National Healthcare Retention & RN Staffing Report states that each 1% increase in nurse turnover costs the average hospital an additional $328,400 [8]. The turnover rate of bedside nurses in hospitals has increased to 17.2%, costing hospitals between $4.4 million and $6.9 million annually [8].

Numerous factors contribute to nurse turnover, including limited promotion opportunities, low salaries, burnout, job dissatisfaction, lack of independence and respect, inadequate staffing and scheduling, and poor doctor-nurse relationships. [7,9,10] The increasing workload and emotional stress, coupled with the lack of support, contribute to burnout, leading to higher turnover rates. [11] Despite efforts by many hospitals to address these issues, nurse turnover rates continue to rise, worsening the global nursing shortage and reducing the efficiency of healthcare systems [12].

Several prediction models have been developed to assess the risk of nurse turnover using various indicators, such as nurse managers’ leadership style, age, working hours, income, and the availability of electronic health records [13–16]. However, the methodological quality and performance of these models remain unclear, making it challenging for hospital managers and policymakers to select the most effective tools for predicting nurse turnover. Therefore, a systematic review is needed to synthesize these models, identify their strengths and weaknesses, and offer clear guidance for improving turnover prediction accuracy. This systematic review aims to critically evaluate existing prediction models for nurse turnover, assess their strengths and limitations, and provide actionable insights that can guide hospital administrators and policymakers in mitigating turnover rates and addressing the global nursing shortage.

### Objectives

The objective of this systematic review is to comprehensively summarize the characteristics, methodologies, and performance metrics of prediction models for nurse turnover.

## Methods and analysis

This systematic review of prediction models for nurse turnover will follow the Checklist for critical Appraisal and data extraction for systematic Reviews of prediction Modelling Studies (CHARMS) [17] and Preferred Reporting Items for Systematic Reviews and Meta-Analysis (PRISMA) guidelines [18]. In addition, this study protocol followed the Preferred Reporting Items for Systematic Review and Meta-Analysis Protocols (PRISMA-P) [19] and has been registered on International Prospective Register of Systematic Reviews (PROSPERO) (CRD42024576727). The PICOS of this systematic review is as followed:

P (Population): Registered nurses, nurse practitioners, and other nursing staff in various healthcare settings, such as hospitals, long-term care facilities, and community care environments.

I (Intervention model): Published prediction models for nurse turnover.

C (Comparator): No competing prediction models will be compared.

O (Outcome): nurse turnover.

S (Setting): This prediction model aims to help medical institutions reduce nurses’ willingness to leave by predicting the risk of nurse turnover.

### Search strategy

A thorough literature search will be carried out in PubMed, Embase, Web of Science, SinoMed, CINAHL, Cochrane Library, China National Knowledge Infrastructure (CNKI), Wanfang Data, and China Science and Technology Journal Database (VIP). All relevant publications published between the creation of these databases and May 31, 2026, will be included in this review.

The search strategy was developed by one author (YJ Huang) based on the research question. And it was peer-reviewed by another author (CY Huang) using the PRESS guideline to ensure its comprehensiveness and precision. Medical Subject Headings (MeSH) and keywords, as well as synonyms related to nurse turnover and prediction models, will be combined into the search strategy. The search technique is shown in Appendix 1. To ensure that all potentially relevant literature is captured, we will also search the references of relevant studies. Gray literature will be identified through the CNKI dissertation database, the ProQuest Dissertations & Theses Global database, and the Wanfang dissertation database. Two independent reviewers will search the databases mentioned above and relevant literature, and disagreements between the two reviewers will be solved through discussion or by consulting a third reviewer. This approach was intended to identify all relevant researches in selected databases, regardless of the original publication language.

### Inclusion criteria and exclusion criteria

Studies that met the following criteria will be included:

- **Population:** Registered nurses, nurse practitioners, and other nursing staff in various healthcare settings, such as hospitals, long-term care facilities, and community care environments.
- **Type of study:** any type of study designed to develop a prediction model for nurse turnover with or without validation, such as cross-sectional study, prospective/retrospective cohort study, nested case-control study, and case-cohort study.
- **Outcomes:** nurse turnover.
- **Types of prediction models:** Prediction models developed using either heuristic or statistical approaches. Heuristic approaches involve selecting and weighing predictors based on expert experience or knowledge (e.g., expert consensus or Delphi technique). Statistical approaches utilize methods like multivariable analysis and machine learning algorithms to identify and weigh significant predictors. Furthermore, the study must report at least one index to show model performance, such as the concordance index (c-index), area under the receiver operating characteristic curve (AUC), Brier score, Hosmer–Lemeshow test, calibration plot, and observed–expected ratio.

Studies that met the following criteria will be excluded from this review:

- Protocol of prediction model.
- Using a single factor to predict nurse turnover.
- Only report turnover intention without measuring actual nurse turnover.
- Conference abstract.
- Unable to get the full text after contacting the authors for one month.

### Study selection process

We will use EndNote X10 (Clarivate Analytics, Philadelphia, USA) to manage the search results. First, all search results from databases will be imported into EndNote X10, and duplicate records will be removed. Two independent reviewers will then screen each title and abstract according to the inclusion and exclusion criteria of this study. For non-Chinese and non-English studies, we will utilize machine translation tools to translate the title and abstract into English or Chinese for the initial eligibility assessment. Full text of the potentially relevant literature identified by at least one reviewer will be obtained to further evaluate whether it meets the inclusion criteria. If the full text is not available online, we will request it by email from the authors. Disagreements will be resolved through discussion or consultation with a third reviewer.

If a non-Chinese and non-English study appears eligible based on the translated abstract, we will obtain the full-text article. For critical appraisal and data extraction, we will proceed as follows: First use machine translation to translate the full text or key sections. For studies deemed to be of high relevance or methodological importance, we will seek additional verification, this may involve consultation with a native speaker or the use of a professional translation service if budget permitting. Any uncertainties in translation that affect eligibility, risk of bias assessment, or data extraction will be clearly documented.

If the full text cannot be reliably assessed after these efforts, the study will be listed as “awaiting assessment” rather than excluded. We will continue to attempt contacting the authors periodically, and all efforts and outcomes will be documented transparently. No study will be excluded solely due to language barriers.

A PRISMA flow diagram will be included in the final systematic review to illustrate the study selection process. In this protocol, a placeholder will be reserved for that figure.

### Data extraction

We will design a form to record the title, first author’s name, country of study, year of publication, language, type of study design, number of centers, characteristics of participants, sample size, outcomes to be predicted, predictors selection, model development method, measures of model performance, calibration method, validation method, funding sources of the included studies. The form was designed based on the study objectives and key points of the CHARMS checklist, while also referring the data extraction tools used in previous published systematic reviews of predictive models. Two independent reviewers will be responsible for extracting the information from the included studies based on the checklist (S2 File). The 11 domains of the CHARMS list (data sources, participants, predictive indicators, etc.) will be extracted to evaluate the applicability and risk of bias of each study. Disagreements will be resolved through discussion or consultation with a third reviewer.

### Assessment of risk of bias

The Prediction Model Risk of Bias Assessment Tool (PROBAST) will be used to assess the quality and risk of the included prediction models by two independent reviewers [20]. The PROBAST specifically designed for studies on prediction models, includes 20 signaling questions divided into four domains: participants, predictors, outcomes, and analysis. Each question can be answered with “yes”, “probably yes”, “no”, “probably no”, or “no information”. If any question within a domain is answered with “no” or “probably no”, that domain is considered at high risk of bias. The overall risk of bias is deemed low only if all domains are judged to be at low risk. Disagreements between the two reviewers will be resolved through discussion or consultation with a third reviewer.

### Data synthesis

A narrative synthesis will be used to present the results of this study, and if the data are sufficiently homogeneous, meta-analyses will be performed. First, we will summarize the characteristics of the included studies, such as study settings, study designs, and participants. Then, descriptive statistics will report the following information about model development and validation: the outcome to be predicted, the number of events, the model development strategy, candidate predictors, the predictor selection method, the final model predictors, the number of events for each variable, the method to address continuous predictors and missing data, model presentation, and model validation strategy. Finally, the indicators for evaluating the discrimination and calibration of prediction models, such as the c-index and Hosmer-Lemeshow test, will be summarized. However, if there is a high statistical heterogeneity between the included prediction models, subgroup analysis will be conducted, if possible, by 1) geographical region; 2) age.

If a meta-analysis is feasible, the following methods will be applied. For discrimination performance, the C-index or AUC will be pooled using a random-effects model after logit transformation to stabilize variance. For calibration, a narrative synthesis will be conducted instead of meta-analysis, given the anticipated heterogeneity in reporting formats (e.g., calibration slopes, observed-to-expected ratios, calibration plots). Statistical heterogeneity will be assessed using the I² statistic and the Cochrane Q test. If I² exceeds 75%, indicating considerable heterogeneity, no pooled estimate will be reported. All analyses will be performed using R software (version 4.2 or later) with the meta and metafor packages. Subgroup analyses will be conducted if possible, based on geographic region and model development method (e.g., logistic regression vs. machine learning). Sensitivity analyses will be performed by excluding studies with high risk of bias as assessed by PROBAST.

### Patient and public involvement

Patients or the public were not involved in the design, or conduct, or reporting, or dissemination plans of our research.

### Ethics and dissemination

Ethics considerations are not applicable to this study because this study is a systematic review protocol of published studies. The results of this study will be published in a peer-review journal.

## Discussion

This study will be the first systematic review to search and evaluate prediction models for nurse turnover. Nurse turnover remains a significant challenge in healthcare settings, impacting patient care, workforce stability, and operational efficiency. [21] By reviewing and assessing existing prediction models, this study seeks to identify the most reliable indicators for predicting nurse turnover, helping healthcare organizations develop effective retention strategies and improve workforce management.

However, this review has several limitations. First, although we designed a comprehensive search strategy without language filters, this review has limitation in terms of language bias, while we used automated translation for screening titles and abstracts in non-Chinese and non-English studies, our ability to thoroughly appraise and extract data from full-text articles without reliable translations was constrained by practical resources. This may be a potential source of bias, and future systematic reviews on this topic would benefit from a formally assembled multilingual team. Second, the included studies may exhibit considerable heterogeneity due to varying healthcare settings, cultural contexts, and methodologies. This variability may affect the generalizability of the prediction models across different environments. Additionally, this review focuses on quantitative prediction models, potentially overlooking qualitative factors influencing nurse turnover, such as emotional or interpersonal workplace aspects. Despite these limitations, the review is expected to provide valuable insights into factors contributing to nurse turnover and offer guidance on developing more accurate, practical prediction models, which may ultimately assist healthcare leaders and policymakers in reducing nurse turnover, improving job satisfaction, and enhancing patient care outcomes across healthcare settings.

## Supporting information

S1 File The PRISMA-P-checklist. (DOCX)

S2 File The CHARMS checklist. (DOCX)

S1 Appendix The search strategy for database. (DOCX)
