# Supplementary material for: Prediction models for nurse turnover: A protocol for a systematic review
Source: PLoS One. 2026 Aug 25;21(8):e0354384. doi: 10.1371/journal.pone.0354384 (PMC13505909; doi:10.1371/journal.pone.0354384)
Supplement: Supplementary file 2 [file pone.0354384.s002.docx]

**Application Instructions for CHARMS Checklist(The CHecklist for critical Appraisal and data extraction for systematic Reviews of prediction Modelling Studies)**

**Table 1**

| **Item** | **Comments and examples** |
| --- | --- |
| **1. Prognostic versus diagnostic prediction model** | Define whether the aim is to review models to predict:   - Future events: prognostic prediction models - Current (disease) status: diagnostic prediction models |
| **2. Intended scope of the review** | Define intended scope of the review and intended purpose of the models reviewed in it. Examples:   - Models to inform physicians’ therapeutic decision making - Models to inform referral to or withholding from invasive diagnostic testing |
| **3. Type of prediction modelling studies** | Define the type of prediction modelling studies to include. Examples of study types :   - Prediction model development without external validation in independent data - Prediction model development with external validation in independent data - External model validation, possibly with model updating |
| **4. Target population to whom the prediction model applies** | Define the target population relevant to the review scope. Examples:   - Women with diagnosed breast cancer - Healthy adult men in the general population |
| **5. Outcome to be predicted** | Define the outcome of interest to be predicted:   - Specific future event, such as a fatal or non-fatal coronary heart disease - Specific diagnostic target disease, such as presence of lung embolism |
| **6. Time span of prediction** | Define over what specific time period the outcome is predicted (prognostic models only). Example:   - Event within a specific time interval, such as event within 3 months, 1 year, or 10 years |
| **7. Intended moment of using the model** | The systematic review may focus on models to be used at a specific moment in time. Examples:   - Models to be used at the moment of diagnosis of a particular disease - Models to be used preoperatively to predict the risk of postoperative complications - Models to be used in asymptomatic adults to detect undiagnosed type 2 diabetes mellitus |

**Table 2**

| **Domain** | **Key items** | **General** | **Applicability** | **Risk of**  **bias** |
| --- | --- | --- | --- | --- |
| **Source of data** | Source of data (e.g., cohort, case-control, randomized trial participants,  or registry data) |  |  |  |
| **Participants** | Participant eligibility and recruitment method (e.g., consecutive participants, location, number of centers, setting, inclusion and exclusion criteria) |  |  |  |
|  | Participant description |  |  |  |
|  | Details of treatments received, if relevant |  |  |  |
|  | Study dates |  |  |  |
| **Outcome(s) to be**  **predicted** | Definition and method for measurement of outcome |  |  |  |
|  | Was the same outcome definition (and method for measurement) used in all patients? |  |  |  |
|  | Type of outcome (e.g., single or combined endpoints) |  |  |  |
|  | Was the outcome assessed without knowledge of the candidate predictors (i.e., blinded)? |  |  |  |
|  | Were candidate predictors part of the outcome (e.g., in panel or consensus diagnosis)? |  |  |  |
|  | Time of outcome occurrence or summary of duration of follow-up |  |  |  |
| **Candidate predictors**  **(or index tests)** | Number and type of predictors (e.g., demographics, patient history, physical examination, additional testing, disease characteristics) |  |  |  |
|  | Definition and method for measurement of candidate predictors |  |  |  |
|  | Timing of predictor measurement (e.g., at patient presentation, at diagnosis, at treatment initiation) |  |  |  |
|  | Were predictors assessed blinded for outcome, and for each other (if relevant)? |  |  |  |
|  | Handling of predictors in the modelling (e.g., continuous, linear, non-linear transformations or categorized) |  |  |  |
| **Sample size** | Number of participants and number of outcomes/events |  |  |  |
|  | Number of outcomes/events in relation to the number of candidate predictors (Events Per Variable) |  |  |  |
| **Missing data** | Number of participants with any missing value (include predictors and outcomes) |  |  |  |
|  | Number of participants with missing data for each predictor |  |  |  |
|  | Handling of missing data (e.g., complete-case analysis, imputation, or other methods) |  |  |  |
| **Model development** | Modelling method (e.g., logistic, survival, neural networks, or machine learning techniques) |  |  |  |
|  | Modelling assumptions satisfied |  |  |  |
|  | Method for selection of predictors for inclusion in multivariable modelling (e.g., all candidate predictors, pre-selection based on unadjusted association with the outcome) |  |  |  |
|  | Method for selection of predictors during multivariable modelling (e.g., full model approach, backward or forward selection) and criteria used (e.g., p-value, Akaike Information Criterion) |  |  |  |
|  | Shrinkage of predictor weights or regression coefficients (e.g., no shrinkage, uniform shrinkage, penalized estimation) |  |  |  |
| **Model performance** | Calibration (calibration plot, calibration slope, Hosmer-Lemeshow test) and Discrimination (C-statistic, D-statistic, log-rank) measures with  confidence intervals |  |  |  |
|  | Classification measures (e.g., sensitivity, specificity, predictive values, net reclassification improvement) and whether a priori cut points were used |  |  |  |
| **Model evaluation** | Method used for testing model performance: development dataset only (random split of data, resampling methods, e.g., bootstrap or cross-validation, none) or separate external validation (e.g., temporal, geographical, different setting, different investigators) |  |  |  |
|  | In case of poor validation, whether model was adjusted or updated (e.g., intercept recalibrated, predictor effects adjusted, or new predictors added) |  |  |  |
| **Results** | Final and other multivariable models (e.g., basic, extended, simplified) presented, including predictor weights or regression coefficients, intercept,  baseline survival, model performance measures (with standard errors or confidence intervals) |  |  |  |
|  | Any alternative presentation of the final prediction models, e.g., sum score, nomogram, score chart, predictions for specific risk subgroups with performance |  |  |  |
|  | Comparison of the distribution of predictors (including missing data) for development and validation datasets |  |  |  |
| **Interpretation and**  **Discussion** | Interpretation of presented models (confirmatory, i.e., model useful for practice versus exploratory, i.e., more research needed) |  |  |  |
|  | Comparison with other studies, discussion of generalizability, strengths and limitations |  |  |  |

*From Moons KG, de Groot JA, Bouwmeester W, et al. Critical appraisal and data extraction for systematic reviews of prediction modelling studies: the CHARMS checklist. PLoS medicine 2014;11(10):e1001744.*
