# Supplementary material for: Prediction models for nurse turnover: A protocol for a systematic review
Source: PLoS One. 2026 Aug 25;21(8):e0354384. doi: 10.1371/journal.pone.0354384 (PMC13505909; doi:10.1371/journal.pone.0354384)
Supplement: Supplementary file 3 [file pone.0354384.s003.docx]

**Appendix 1 The search strategy for database**

| **Database** | **Number** | **Searches** |
| --- | --- | --- |
| **Pubmed** | **#1** | "Nurses"[Mesh] OR "Nursing"[Mesh] OR "Nursing staff"[Mesh] OR nurse* [Title/Abstract] OR "nursing" [Title/Abstract] |
|  | **#2** | "Personnel Turnover"[Mesh] OR turnover* [Title/Abstract] OR attrition [Title/Abstract] OR "job retention" [Title/Abstract] OR "intent to leave" [Title/Abstract] OR "personnel retention" [Title/Abstract] |
|  | **#3** | "Models, Statistical"[Mesh] OR "Forecasting"[Mesh] OR "Machine Learning"[Mesh] OR "Risk Assessment"[Mesh] OR "risk prediction" [Title/Abstract] OR algorithm* [Title/Abstract] OR "data mining” [Title/Abstract] OR "artificial intelligence" [Title/Abstract] OR predict*[Title/Abstract] OR prognos* [Title/Abstract] OR model* [Title/Abstract] |
|  | **#4** | #1 AND #2 AND #3 |
| **Embase** | **#1** | 'nurse'/exp OR 'nursing staff'/exp OR nurse*:ti,ab,kw OR 'nursing staff ':ti,ab,kw |
|  | **#2** | 'personnel turnover'/exp OR turnover*:ti,ab,kw OR attrition:ti,ab,kw OR 'job retention':ti,ab,kw OR 'intent to leave':ti,ab,kw OR 'personnel retention':ti,ab,kw |
|  | **#3** | 'models, statistical'/exp OR 'forecasting'/exp OR 'machine learning'/exp OR 'risk assessment'/exp OR 'artificial intelligence'/exp OR 'data mining'/exp OR 'algorithm'/exp OR 'risk prediction':ti,ab,kw OR algorithm*:ti,ab,kw OR 'data mining':ti,ab,kw OR 'artificial intelligence':ti,ab,kw OR predict*:ti,ab,kw OR prognos*:ti,ab,kw OR model*:ti,ab,kw |
|  | **#4** | #1 AND #2 AND #3 |
| **Web of Science** | **#1** | (TS=(nursing staff)) OR TS=(nurse*) |
|  | **#2** | ((((TS=(turnover)) OR TS=(attrition)) OR TS=(job retention)) OR TS=(intent to leave)) OR TS=(personnel retention) |
|  | **#3** | ((((((((((TS=(Models, Statistical)) OR TS=(Forecasting)) OR TS=(Machine Learning)) OR TS=(Risk Assessment)) OR TS=(risk prediction)) OR TS=(algorithm)) OR TS=(data mining)) OR TS=(artificial intelligence)) OR TS=(predict*)) OR TS=(prognos*)) OR TS=(model*) |
|  | **#4** | #1 AND #2 AND #3 |
| **CINAHL** | **S1** | MH "Nurses" OR TI nurse* OR AB nurse* OR KW nurse* |
|  | **S2** | MH "Personnel Turnover" OR TI turnover* OR AB turnover* OR KW turnover* OR TI attrition OR AB attrition OR KW attrition OR TI "intent to leave" OR AB "intent to leave" OR KW "intent to leave" OR TI "job retention" OR AB "job retention" OR KW "job retention" OR TI "personnel retention" OR AB "personnel retention" OR KW "personnel retention" |
|  | **S3** | MH "Statistical Models" OR MH "Forecasting" OR MH "Machine Learning" OR MH "Risk Assessment" OR TI "risk prediction" OR AB "risk prediction" OR KW "risk prediction" OR TI algorithm* OR AB algorithm* OR KW algorithm* OR TI "data mining" OR AB "data mining" OR KW "data mining" OR TI "artificial intelligence" OR AB "artificial intelligence" OR KW "artificial intelligence" OR TI predict* OR AB predict* OR KW predict* OR TI prognos* OR AB prognos* OR KW prognos* OR TI model* OR AB model* OR KW model* |
|  | **S4** | S1 AND S2 AND S3 |
| **Cochrane Library** | **#1** | (nurse):ti,ab,kw OR (nursing staff):ti,ab,kw |
|  | **#2** | (turnover):ti,ab,kw OR (attrition):ti,ab,kw OR (job retention):ti,ab,kw OR (intent to leave):ti,ab,kw OR (personnel retention):ti,ab,kw |
|  | **#3** | (models,statistical):ti,ab,kw OR (forecasting):ti,ab,kw OR (machine learning):ti,ab,kw OR (risk assessment):ti,ab,kw OR (artificial intelligence):ti,ab,kw OR (data mining):ti,ab,kw OR (algorithm):ti,ab,kw OR (predictive model):ti,ab,kw OR (prediction model):ti,ab,kw OR (risk prediction):ti,ab,kw OR (prognostic model):ti,ab,kw OR (algorithm):ti,ab,kw |
|  | **#4** | #1 AND #2 AND #3 |
| **Wanfang Data** | ( (title:(nurse) or key word:(nurse) or abstract:(nurse)) or (title:(nursing) or key word:(nursing) or abstract:(nursing)) ) and ( (title:(predictive model) or key word:(predictive model) or abstract:(predictive model)) or (title:(forecasting) or key word:(forecasting) or abstract:(forecasting)) or (title:(risk forecasting) or key word:(risk forecasting) or abstract:(risk forecasting)) or (title:(machine learning) or key word:(machine learning) or abstract:(machine learning)) or (title:(artificial intelligence) or key word:(artificial intelligence) or abstract:(artificial intelligence)) or (title:(algorithm) or key word:(algorithm) or abstract:(algorithm)) or (title:(data mining) or key word:(data mining) or abstract:(data mining)) or (title:(prognostic model) or key word:(prognostic model) or abstract:(prognostic model)) ) and ( (title:(turnover) or key word:(turnover) or abstract:(turnover)) or (title:( intention to leave) or key word:( intention to leave) or abstract:( intention to leave)) or (title:(attrition) or key word:(attrition) or abstract:(attrition)) ) | |
| **China Science and Technology Journal Database (VIP)** | ((M=(predictive model) OR M=(forecasting) OR M=(risk forecasting) OR M=(machine learning) OR M=(artificial intelligence) OR M=(algorithm) OR M=(data mining) OR M=(prognostic model)) OR (R=(predictive model) OR R=(forecasting) OR R=(risk forecasting) OR R=(machine learning) OR R=(artificial intelligence) OR R=(algorithm) OR R=(data mining) OR R=(prognostic model))) AND ((M=(nurse) OR M=(nursing staff) OR M=(nursing)) OR (R=(nurse) OR R=(nursing staff) OR R=(nursing))) AND ((M=(turnover) OR M=( intention to leave) OR M=(attrition)) OR (R=(turnover) OR R=(intention to leave) OR R=(attrition))) | |
| **China National Knowledge Infrastructure (CNKI)** | (TI='nurse' OR TI='nursing staff' OR TI='nursing' OR AB='nurse' OR AB='nursing staff' OR AB='nursing') AND (TI='predictive model' OR TI='forecasting' OR TI='risk forecasting' OR TI='machine learning' OR TI='artificial intelligence' OR TI='algorithm' OR TI='data mining' OR TI='prognostic model' OR AB='predictive model' OR AB='forecasting' OR AB='risk forecasting' OR AB='machine learning' OR AB='artificial intelligence' OR AB='algorithm' OR AB='data mining' OR AB='prognostic model') AND (TI='turnover' OR TI='intention to leave' OR TI='attrition' OR AB='turnover' OR AB='intention to leave' OR AB='attrition') | |
| **Sinomed** | **1** | "nurse"[Common Fields: Intelligent] OR "nursing staff"[Common Fields: Intelligent] OR "nursing"[Common Fields: Intelligent] |
|  | **2** | "predictive model"[Common Fields: Intelligent] OR "forecasting"[Common Fields: Intelligent] OR "risk forecasting"[Common Fields: Intelligent] OR "machine learning"[Common Fields: Intelligent] OR "artificial intelligence"[Common Fields: Intelligent] OR "algorithm"[Common Fields: Intelligent] OR "data mining"[Common Fields: Intelligent] OR "prognostic model"[Common Fields: Intelligent] |
|  | **3** | "turnover"[Common Fields: Intelligent] OR "intention to leave"[Common Fields: Intelligent] OR "attrition"[Common Fields: Intelligent] |
|  | **4** | (#3) AND (#2) AND (#1) |
